# Supplementary figures and images for: Elucidating multi-input processing 3-node gene regulatory network topologies capable of generating striped gene expression patterns
Source: PLoS Comput Biol. 2022 Feb 14;18(2):e1009704. doi: 10.1371/journal.pcbi.1009704 (PMC8880922; doi:10.1371/journal.pcbi.1009704)

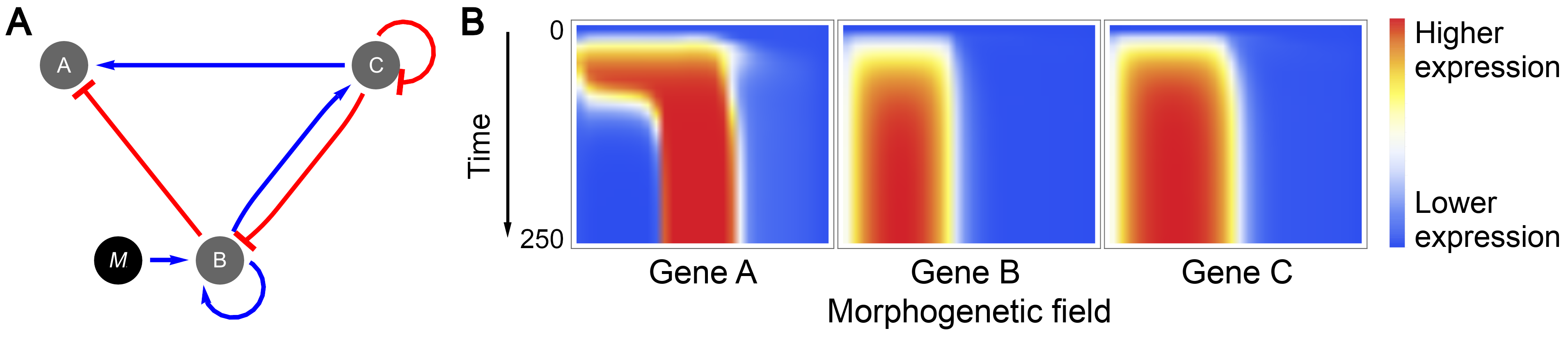

Supplement: S1 Fig — (A) Gene regulatory network displaying the topology number 33. (B) Spatiotemporal expression profile of the gene regulatory network shown in (A). (TIF) [file pcbi.1009704.s001.tif]

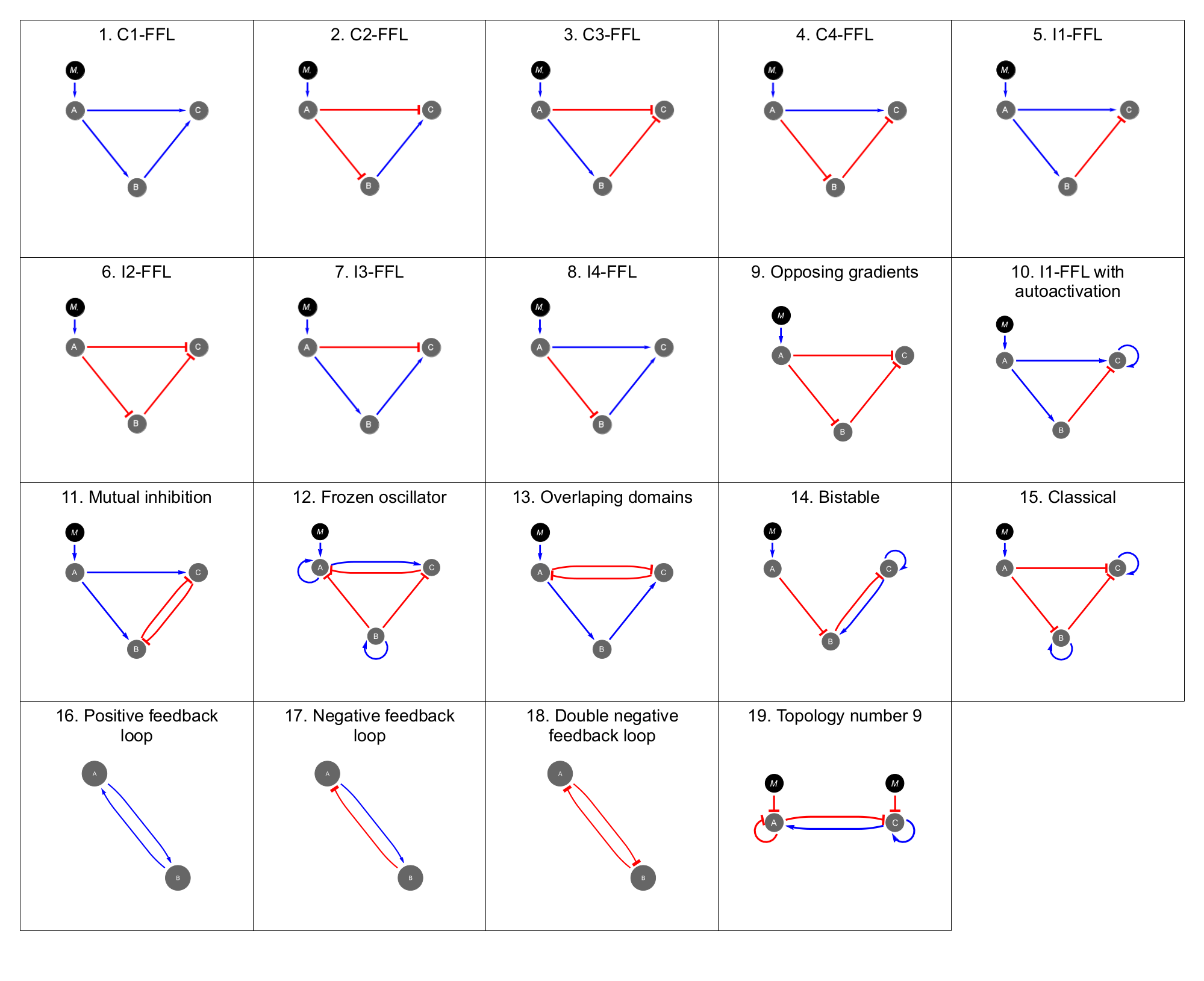

Supplement: S2 Fig — These are subgraphs that have been reported in previous studies as networks involved in morphogenesis and development [28, 32, 35]. These were used to calculate the subgraph profile and the results reported in S1 Table. (TIF) [file pcbi.1009704.s002.tif]

A

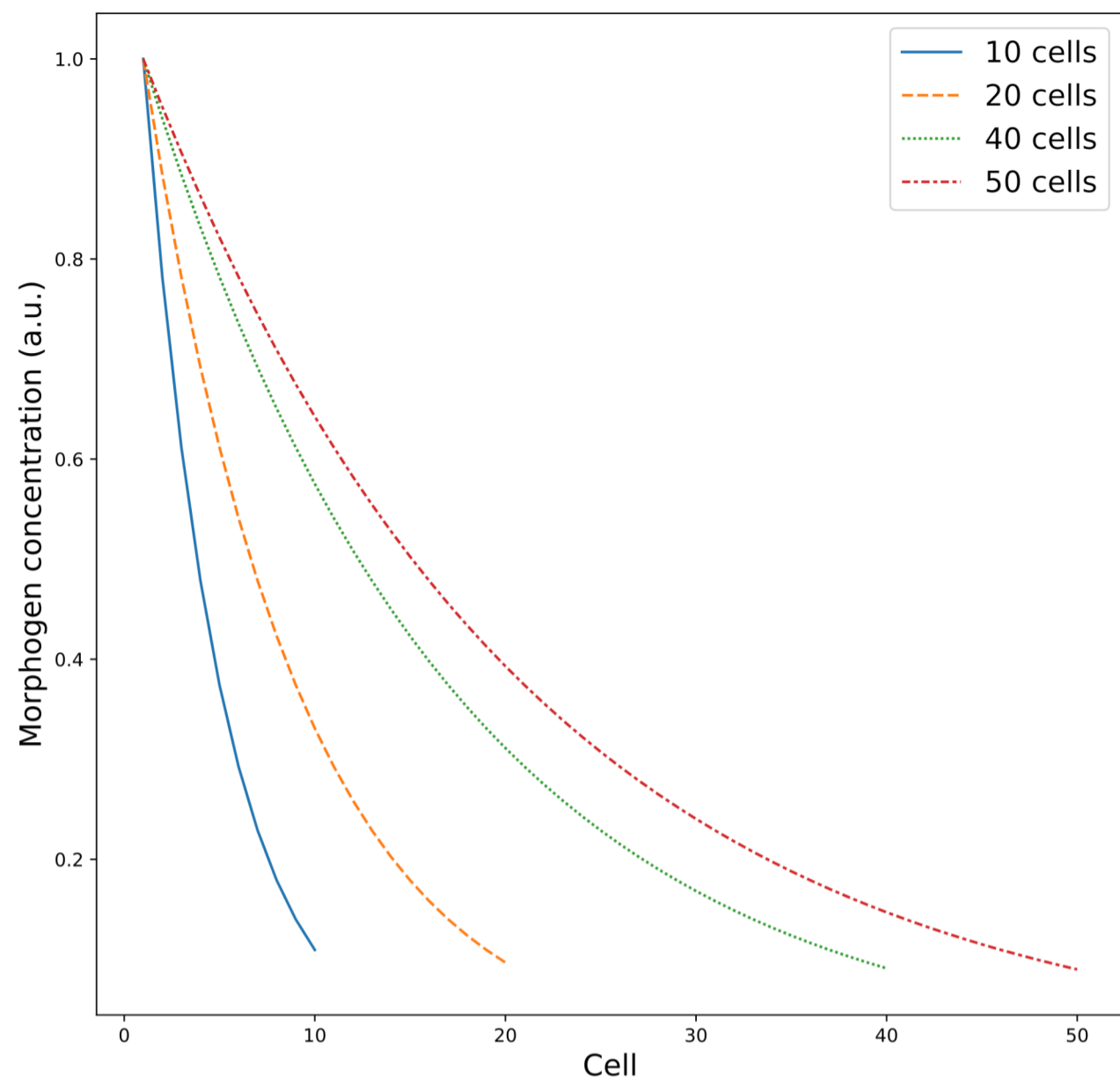

B

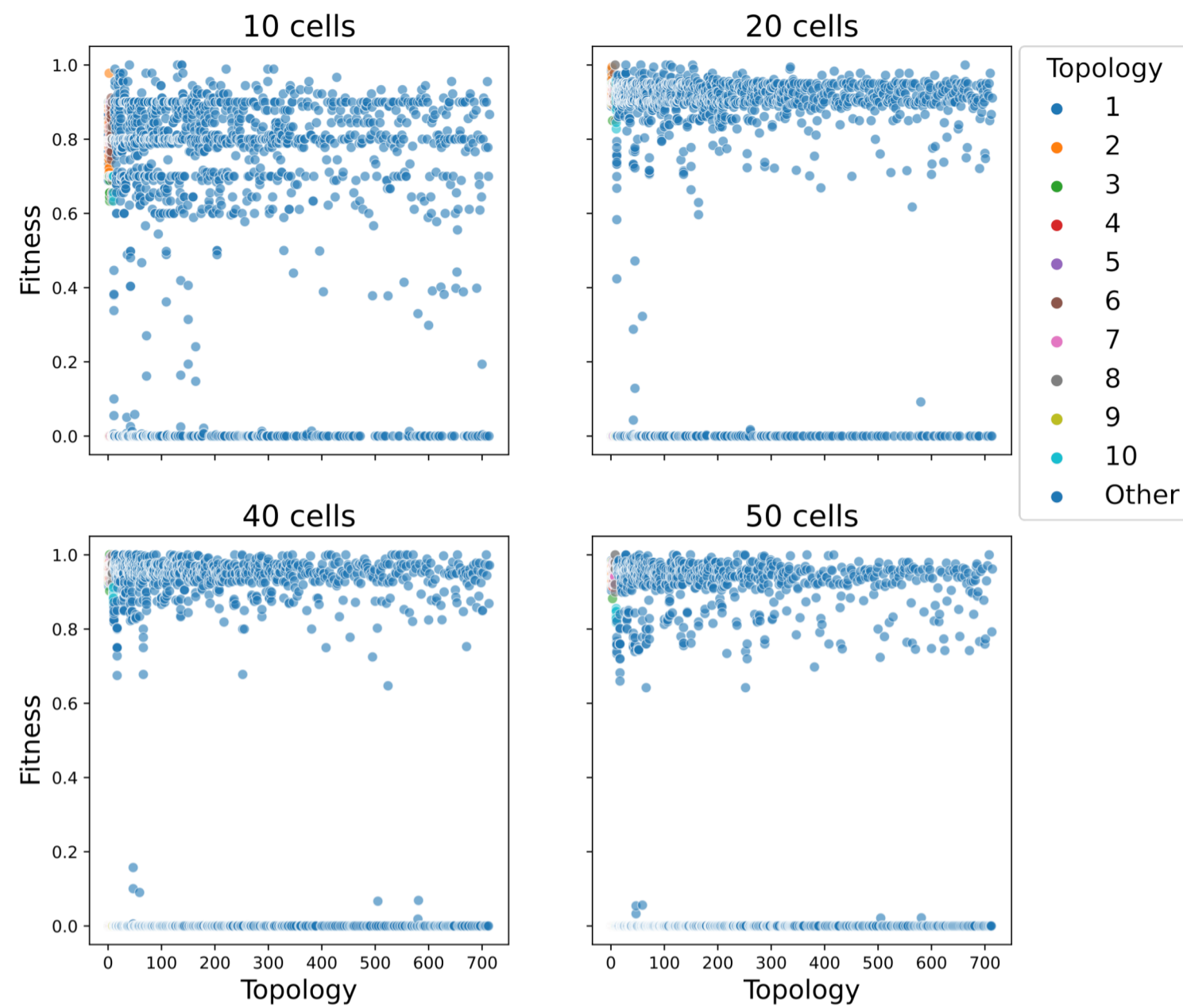

C

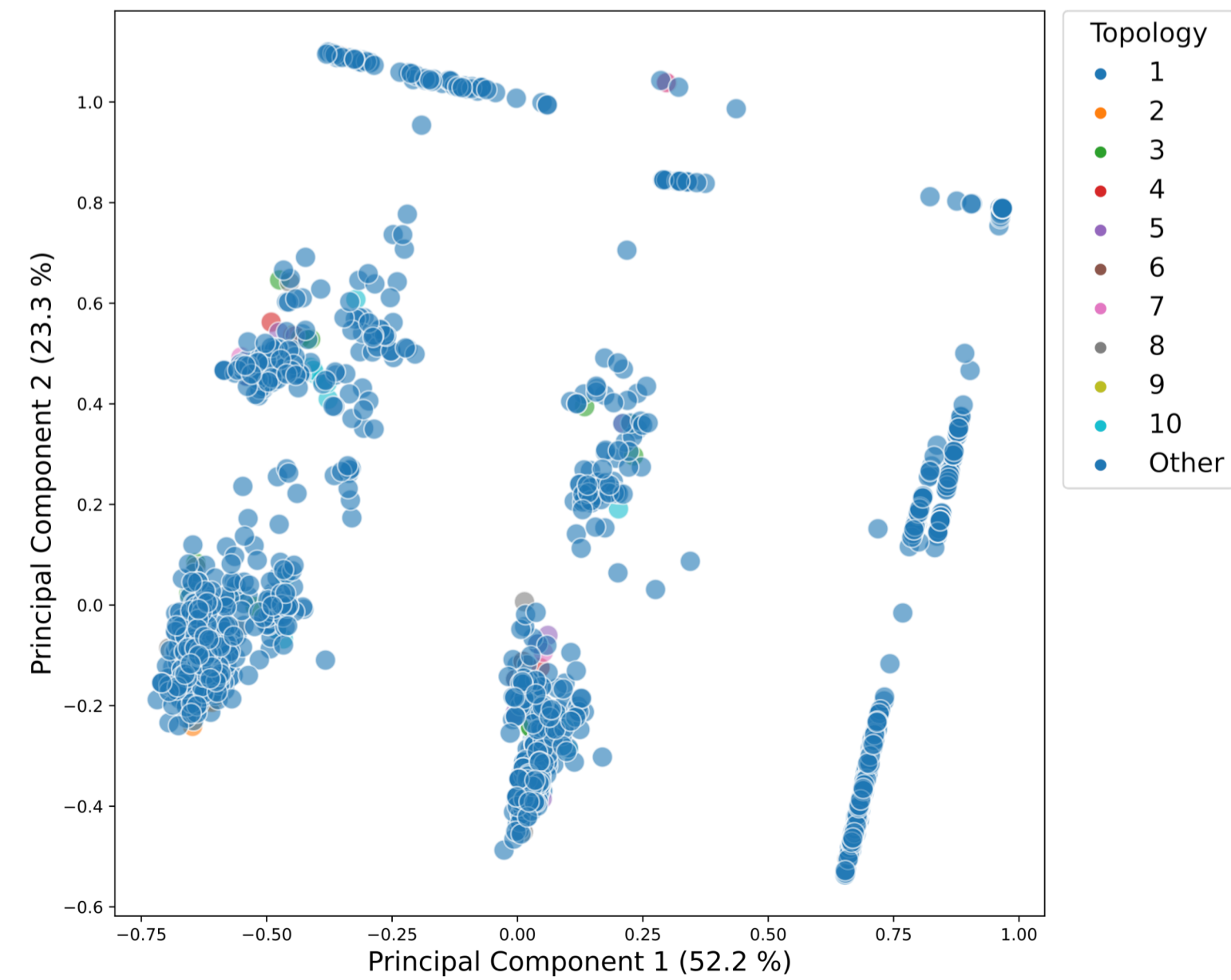

D

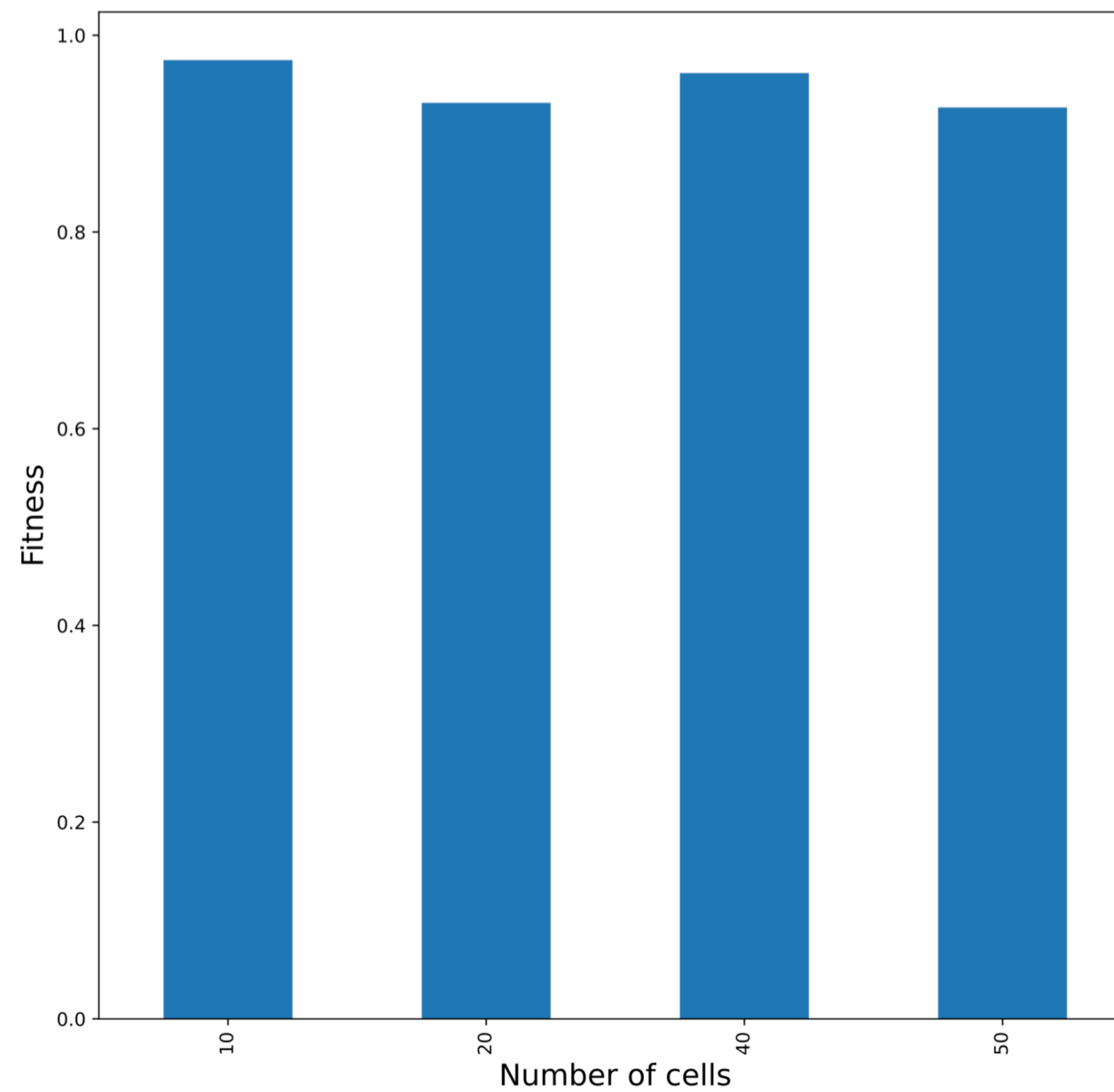

Supplement: S3 Fig — (A) Morphogen gradients of fields with 10, 20, 40 and 50 cells. These morphogen gradients were set up so that the morphogen concentration in the first cell and in the last cell were constant and only one morphogen gradient was considered at a time depending on the number of cells in the morphogenetic field. (B) Fitness of the GRNs by topology in each one of the morphogenetic fields. (C) Principal Component Analysis of fitness by morphogenetic field size. Although principal components separate GRNs in clusters, not all the GRNs with the same topology are located in the same group. (D) Average fitness of GRNs evolved in different morphogenetic field sizes. (PDF) [file pcbi.1009704.s003.pdf]

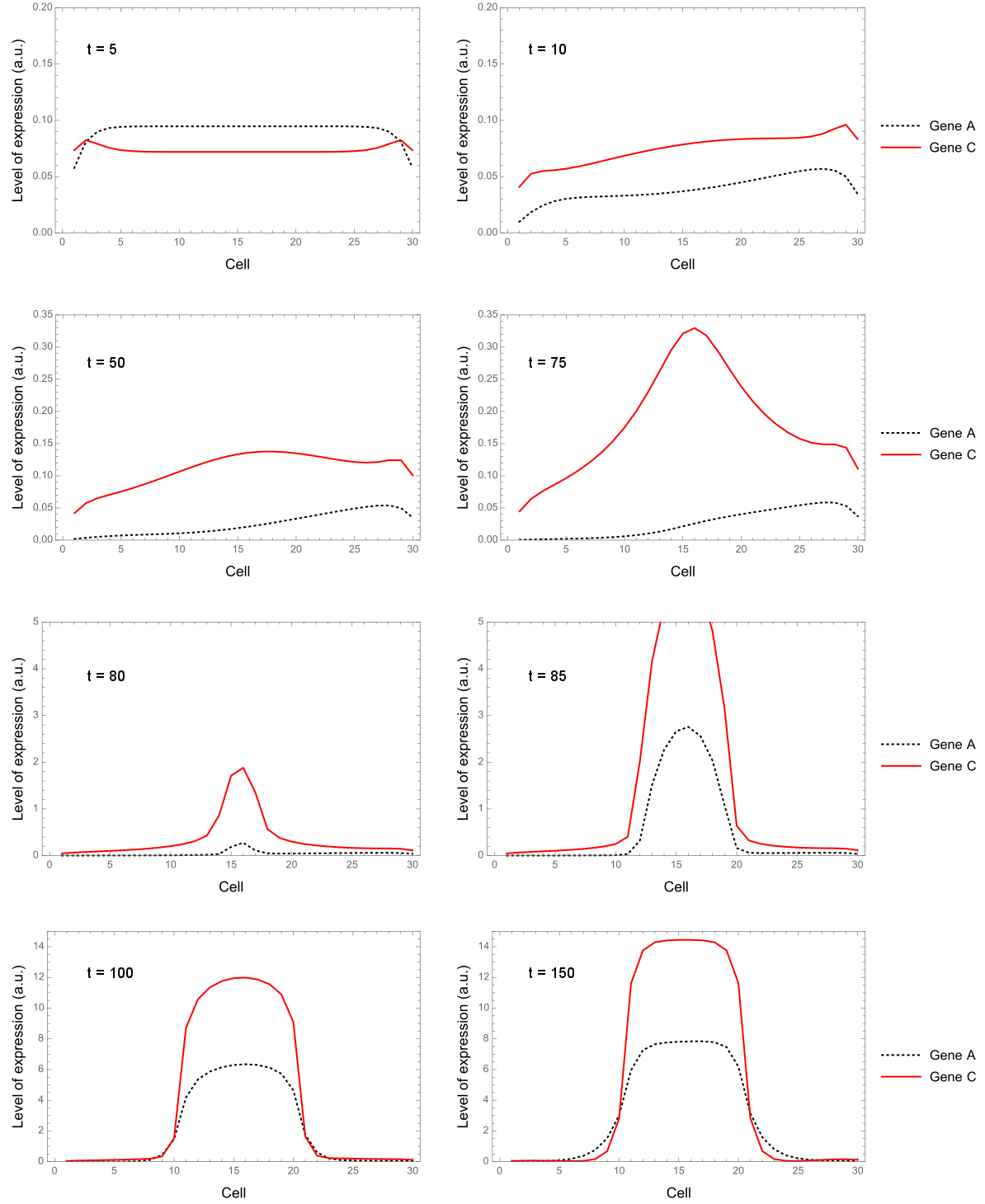

Supplement: S5 Fig — The dynamics of expression is shown from t = 5 to t = 150, where this topology reaches its steady state expression. The red line represents the expression level of the gene C along the morphogenetic field, whereas the dotted line represents the expression level of gene A. The striped pattern of gene expression can be seen for both genes since t = 80. (TIF) [file pcbi.1009704.s005.tif]

**A**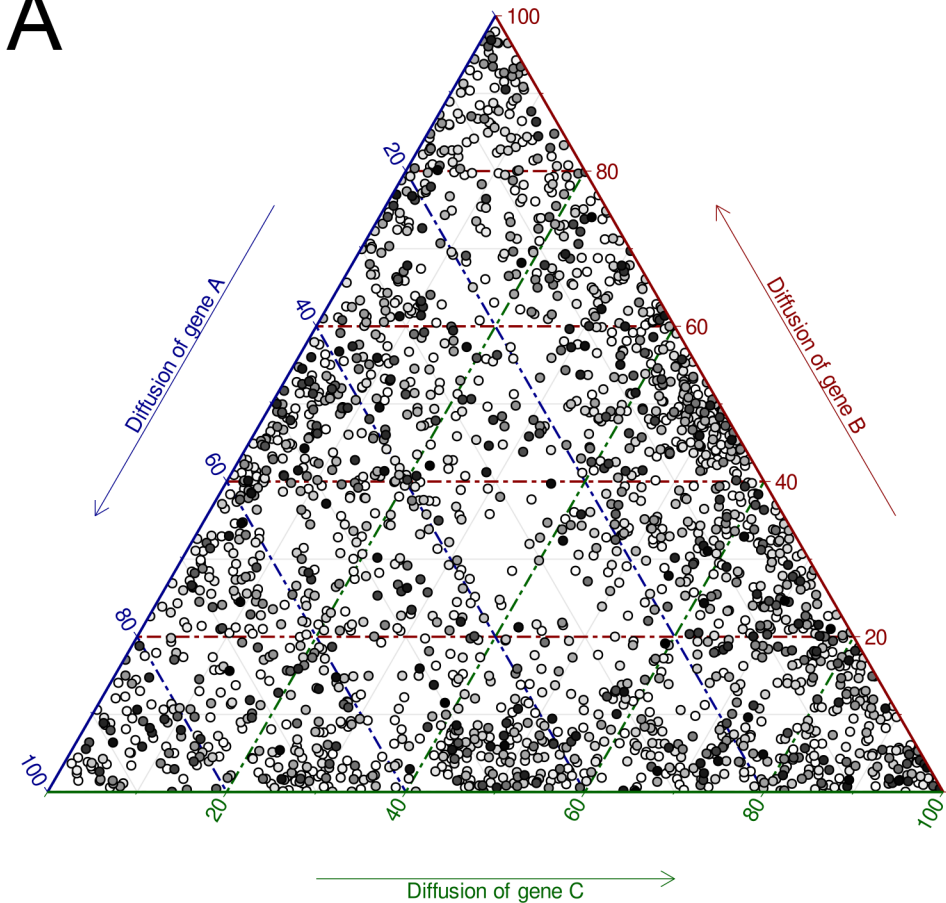**B**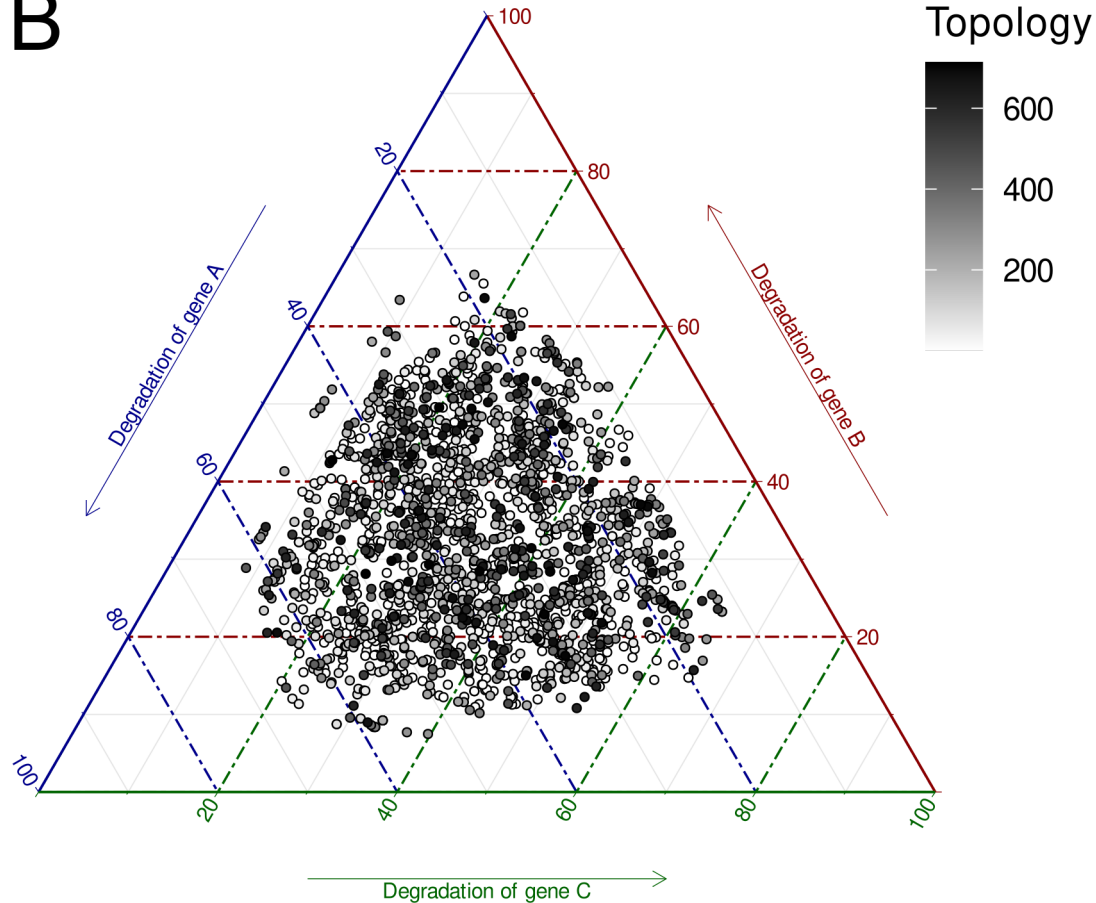

Supplement: S7 Fig — (A) Ternary plot showing the combination of diffusion parameters for genes A, B and C. (B) Ternary plot showing the combination of degradation parameters for genes A, B and C. The color gradient that goes from topology 1 in white to topology 714 in black shows no distinguishable pattern. (PDF) [file pcbi.1009704.s007.pdf]
