# Supplementary material for: Elucidating multi-input processing 3-node gene regulatory network topologies capable of generating striped gene expression patterns
Source: PLoS Comput Biol. 2022 Feb 14;18(2):e1009704. doi: 10.1371/journal.pcbi.1009704 (PMC8880922; doi:10.1371/journal.pcbi.1009704)
Supplement: S4 Fig — The dynamics of expression is shown from t = 1 to t = 30 for the eight most abundant topologies. The red line represents the expression level of the gene C along the morphogenetic field, the blue line represents the expression level of gene B and the dotted line represents the expression level of gene A. (PDF) [file pcbi.1009704.s004.pdf]

Topology 1

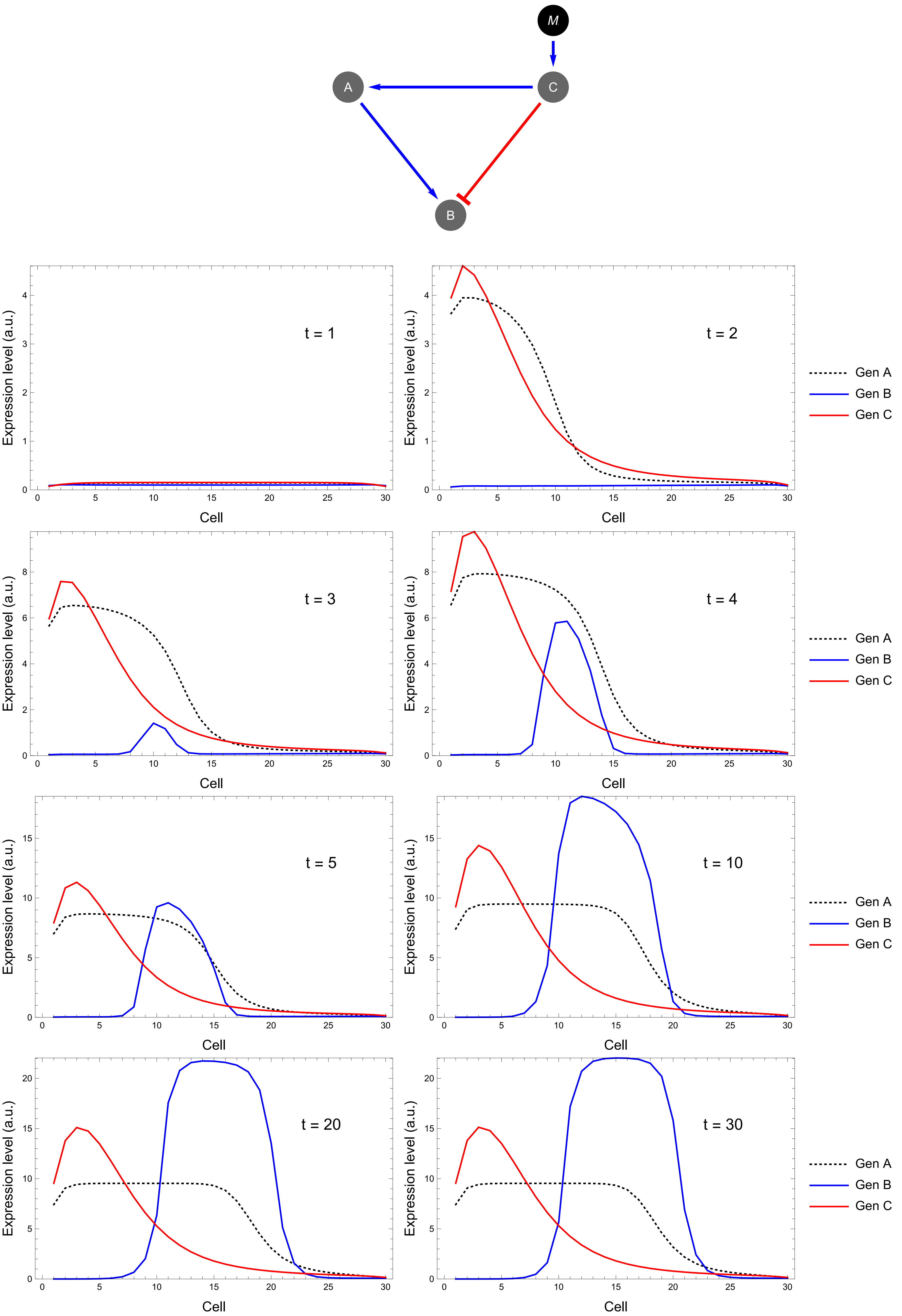

M
A
C
B
t = 1
t = 2
t = 3
t = 4
t = 5
t = 10
t = 20
t = 30
Expression level (a.u.)
Cell
Gen A
Gen B
Gen C

Topology 2

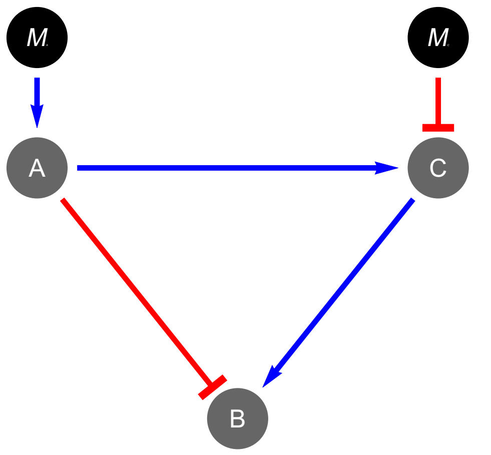
M
M
A
C
B

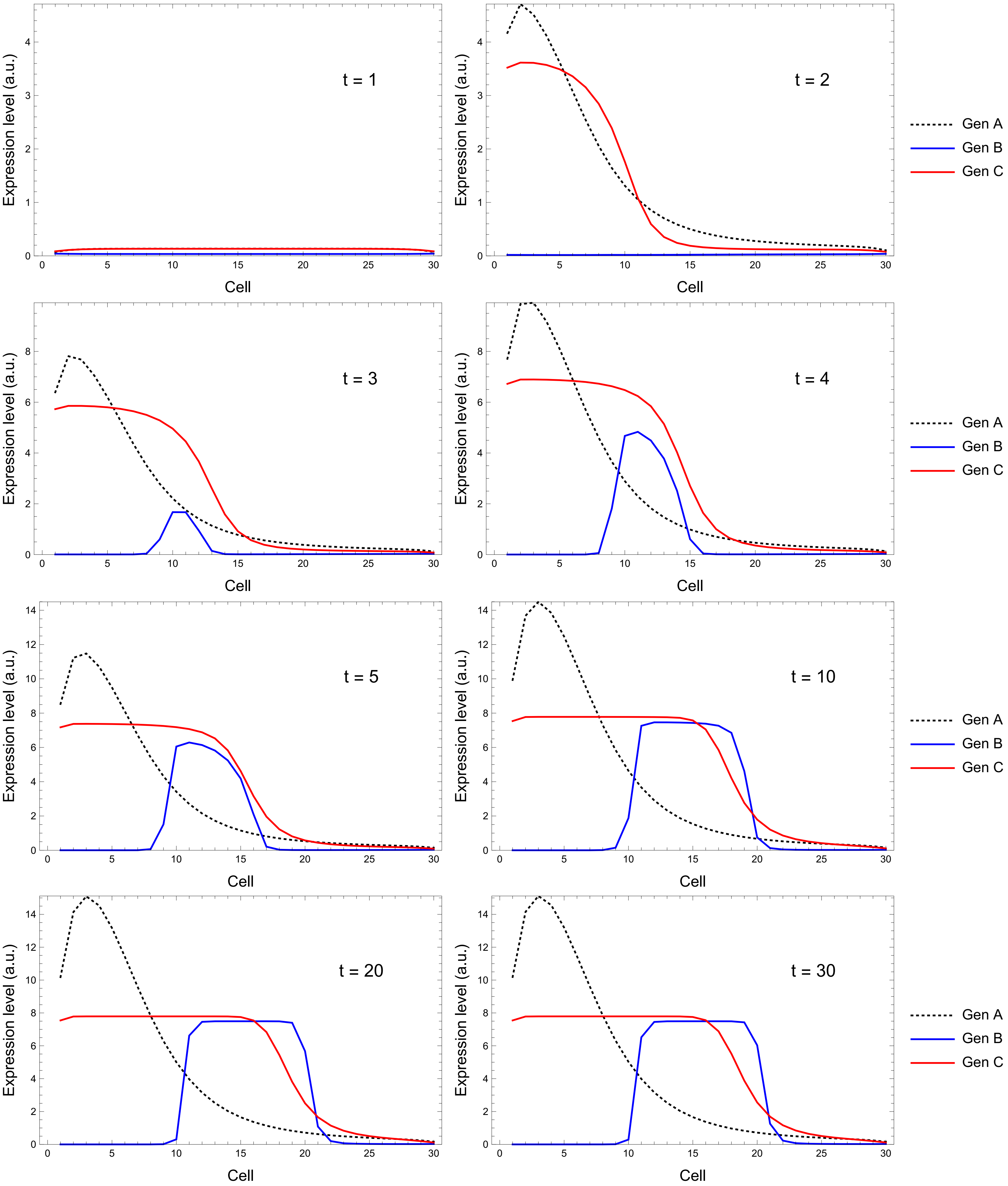
t = 1
t = 2
t = 3
t = 4
t = 5
t = 10
t = 20
t = 30
Expression level (a.u.)
Cell
Gen A
Gen B
Gen C

Topology 3

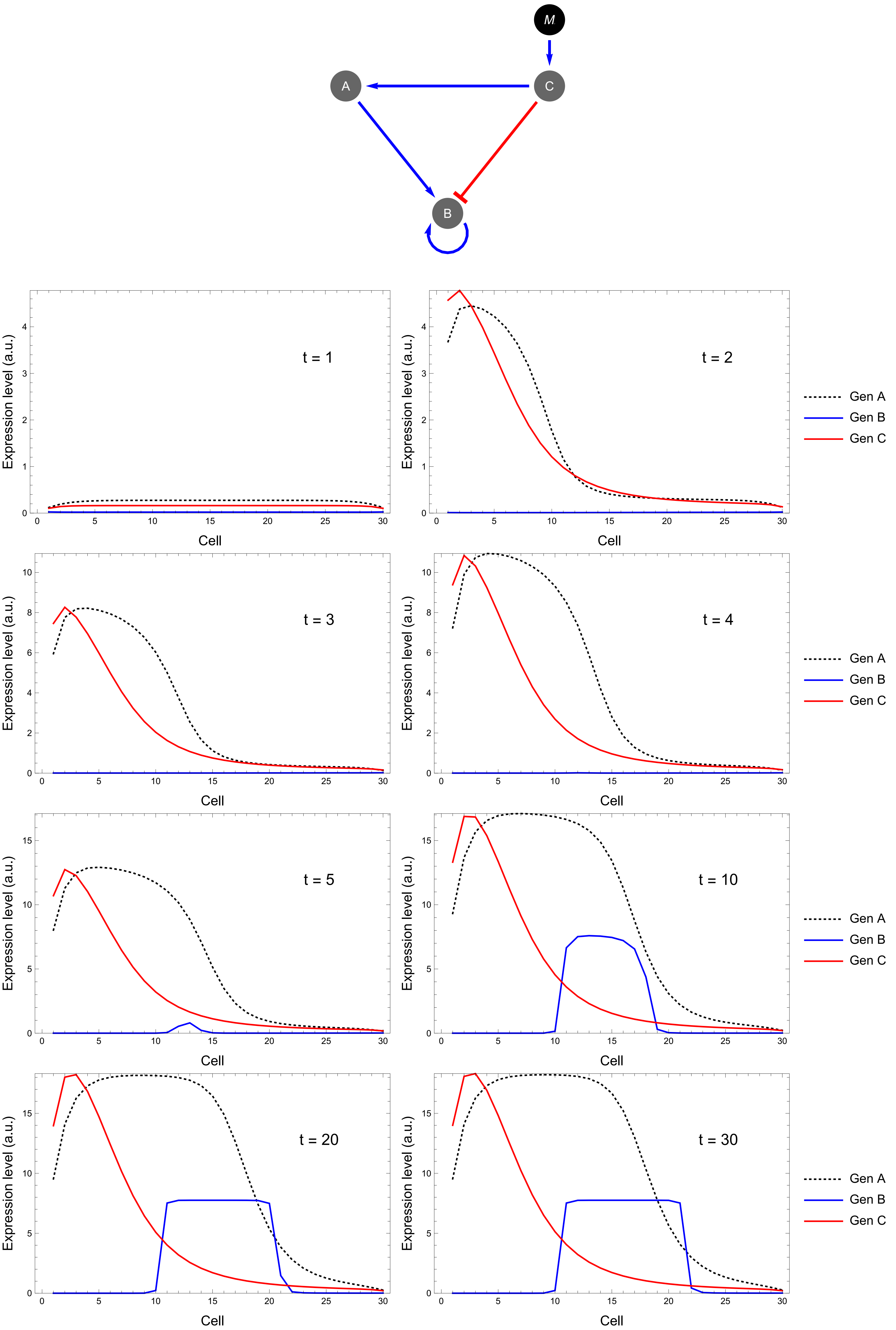
M
A
C
B
t = 1
t = 2
t = 3
t = 4
t = 5
t = 10
t = 20
t = 30
Expression level (a.u.)
Cell
Gen A
Gen B
Gen C

Topology 4

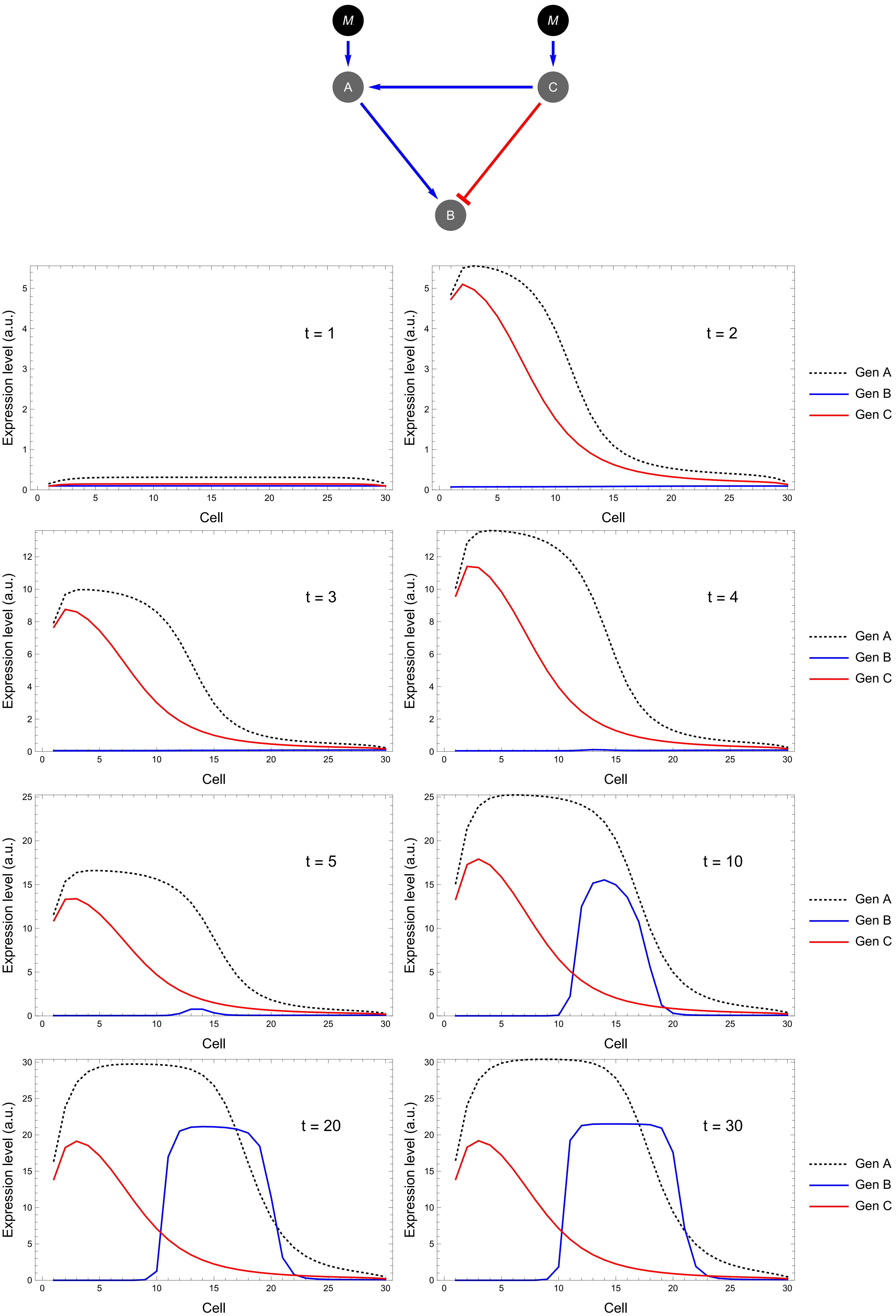

M
M
A
C
B
t = 1
t = 2
t = 3
t = 4
t = 5
t = 10
t = 20
t = 30
Expression level (a.u.)
Cell
Gen A
Gen B
Gen C

Topology 5

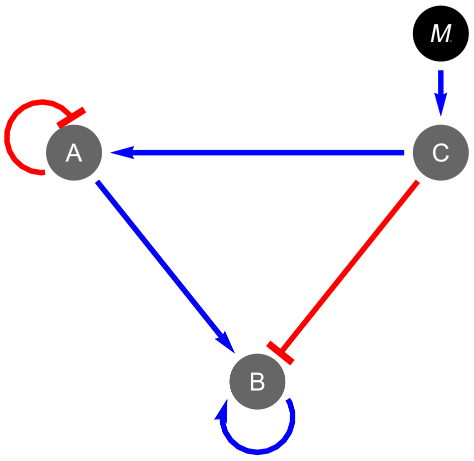
M
A
B
C

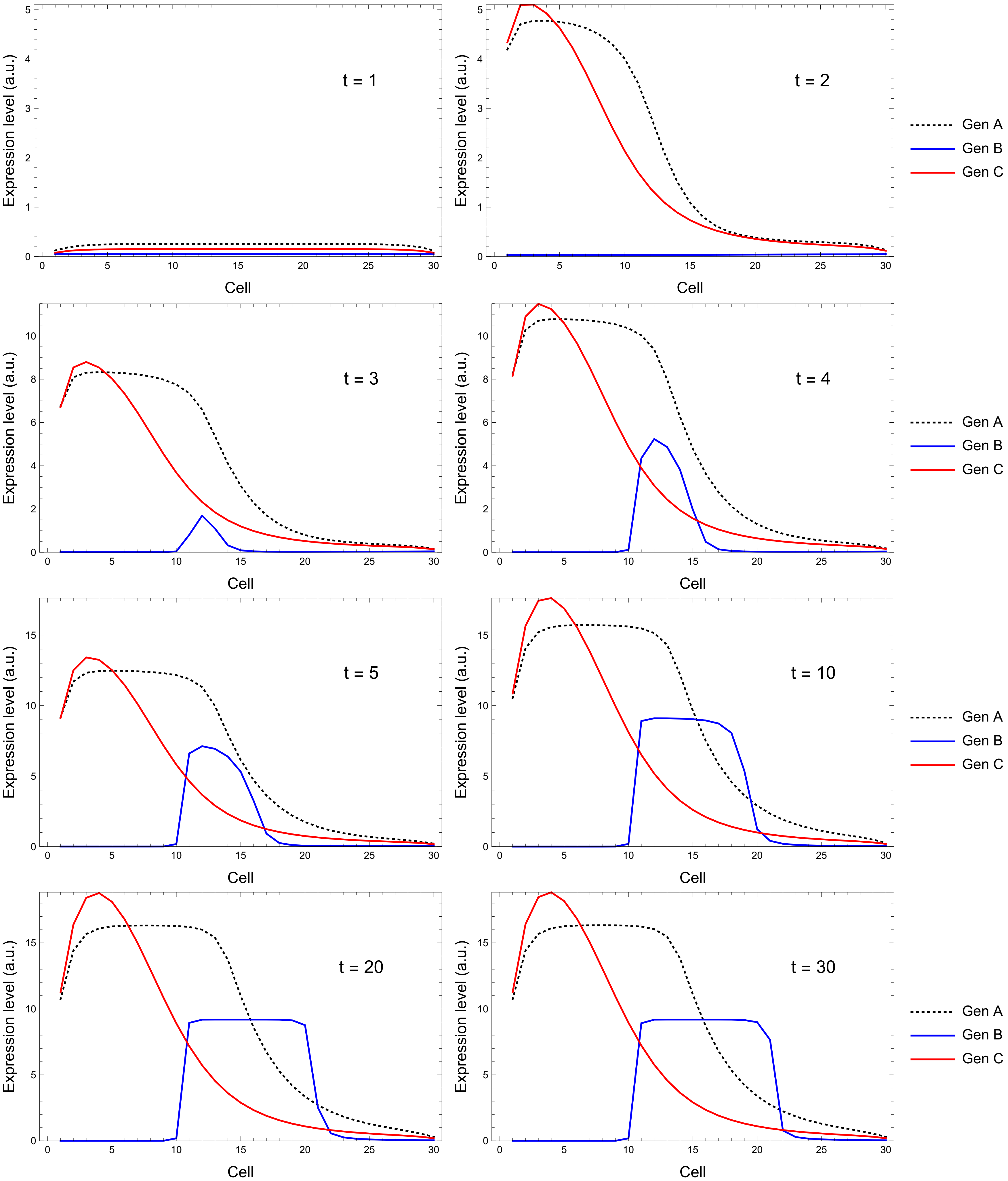
t = 1
t = 2
t = 3
t = 4
t = 5
t = 10
t = 20
t = 30
Expression level (a.u.)
Cell
Gen A
Gen B
Gen C

Topology 6

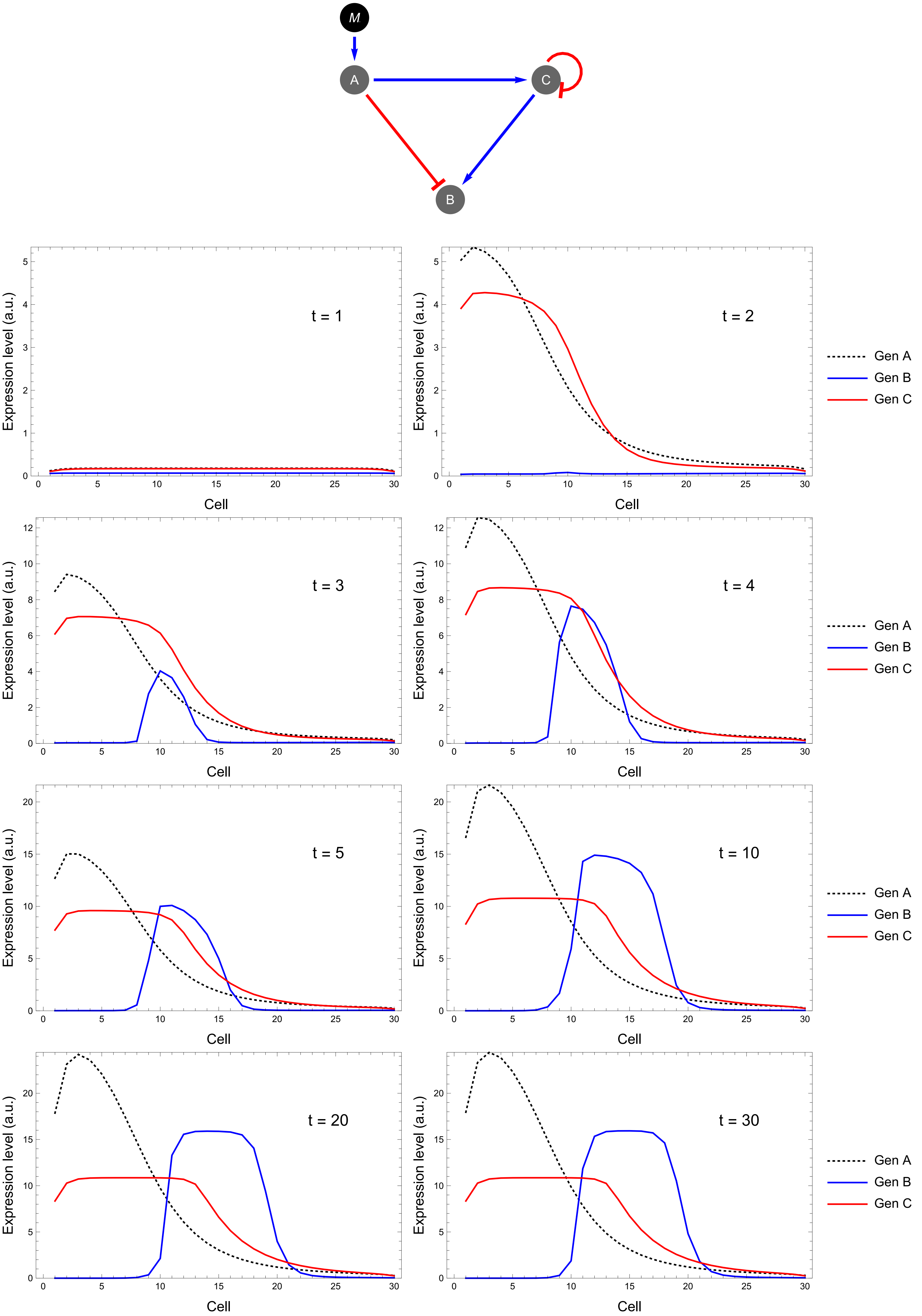
M
A
B
C
t = 1
t = 2
t = 3
t = 4
t = 5
t = 10
t = 20
t = 30
Expression level (a.u.)
Cell
Gen A
Gen B
Gen C

Topology 7

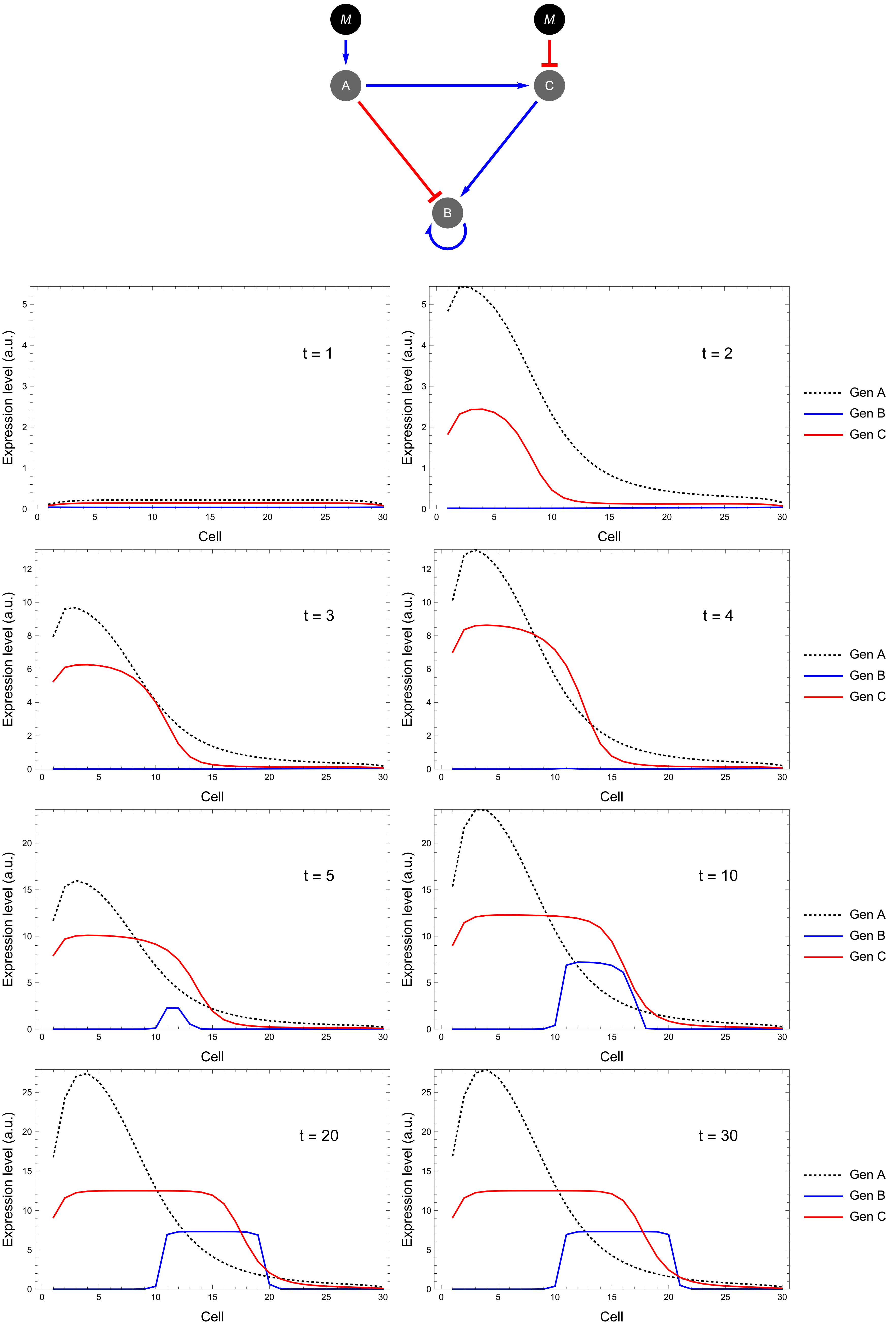
M
M
A
C
B
t = 1
t = 2
t = 3
t = 4
t = 5
t = 10
t = 20
t = 30
Expression level (a.u.)
Cell
Gen A
Gen B
Gen C

Topology 8

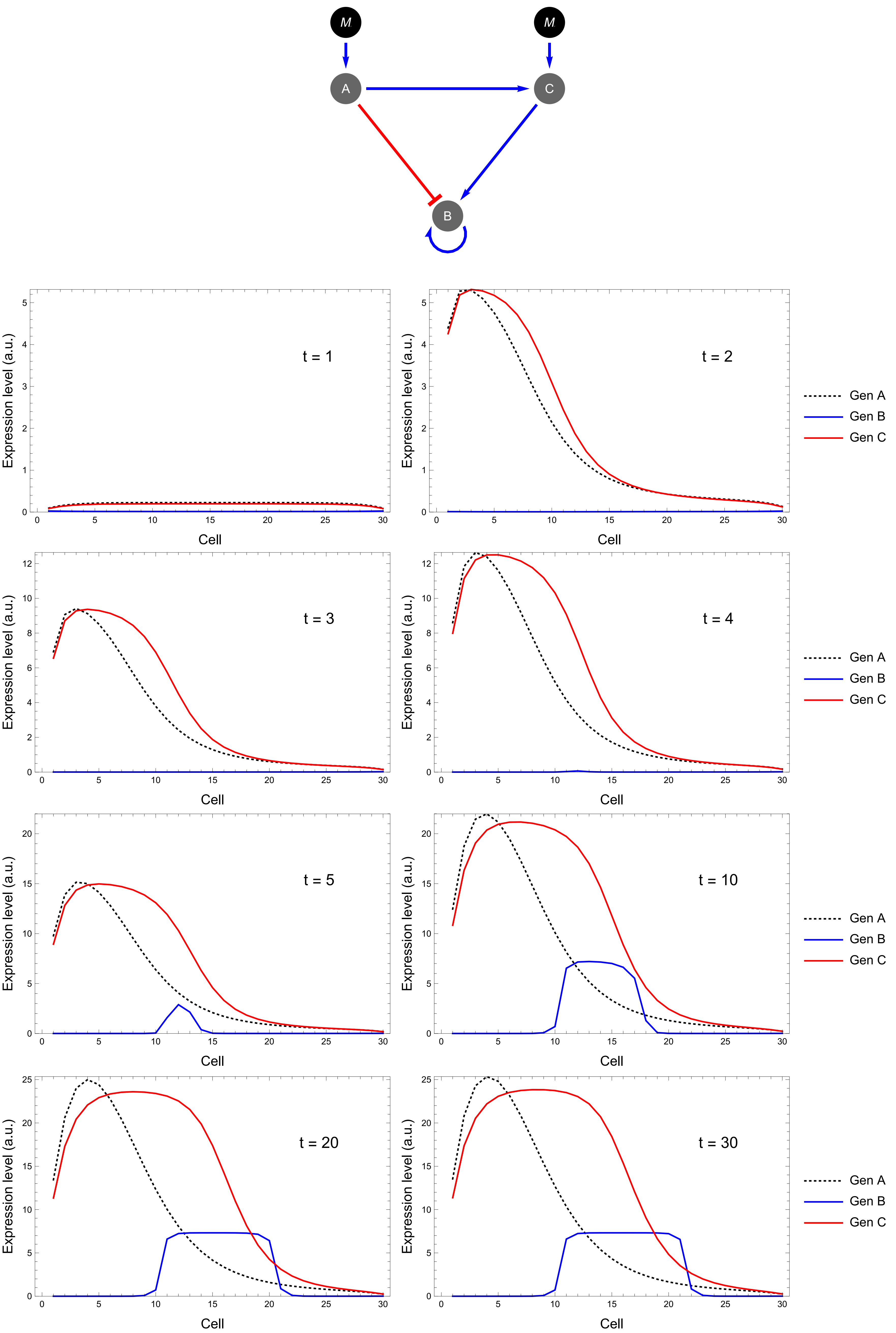

M
M
A
C
B
t = 1
t = 2
t = 3
t = 4
t = 5
t = 10
t = 20
t = 30
Expression level (a.u.)
Cell
Gen A
Gen B
Gen C
